# A New Classification System for Postinterventional Cerebral Hyperdensity: The Influence on Hemorrhagic Transformation and Clinical Prognosis in Acute Stroke

**DOI:** 10.1155/2021/6144304

**Published:** 2021-11-23

**Authors:** Yuan Shao, Yuyun Xu, Yumei Li, Xuehua Wen, Xiaodong He

**Affiliations:** Department of Radiology, Zhejiang Provincial People's Hospital, Affiliated People's Hospital, Hangzhou Medical College, Hangzhou, Zhejiang, China

## Abstract

**Background:**

Postinterventional cerebral hyperdensity (PCHD) is commonly seen in acute ischemic patients after mechanical thrombectomy. We propose a new classification of PCHD to investigate its correlation with hemorrhagic transformation (HT). The clinical prognosis of PCHD was further studied.

**Methods:**

Data from 189 acute stroke patients were analyzed retrospectively. According to the European Cooperative Acute Stroke Study criteria (ECASS), HT was classified as hemorrhagic infarction (HI-1 and HI-2) and parenchymal hematoma (pH-1 and pH-2). Referring to the classification of HT, PCHD was classified as PCHD-1, PCHD-2, PCHD-3, and PCHD-4. The prognosis included early neurological deterioration (END) and the modified Rankin Scale (mRS) score at 3 months.

**Results:**

The incidence of HT was 14.8% (12/81) in the no-PCHD group and 77.8% (84/108) in the PCHD group. PCHD was highly correlated with HT (*r* = 0.751, *p* < 0.01). After stepwise regression analysis, PCHD and the National Institutes of Health Stroke Scale (NIHSS) score at admission were found to be independent factors for END (*p* < 0.001, *p* = 0.015, respectively). The area of curves (AUC) of PCHD, the NIHSS at admission, and the combined model were 0.810, 0.667, and 0.832, respectively. The optimal diagnostic cutoff of PCHD for END was PCHD > 2. PCHD, the NIHSS score at admission, and good vascular recanalization (VR) were independently associated with 3-month mRS (all *p* < 0.05). The AUC of PCHD, the NIHSS at admission, good VR, and the combined model were 0.779, 0.733, 0.565, and 0.867, respectively. And the best cutoff of PCHD for the mRS was PCHD > 1.

**Conclusion:**

The relationship of PCHD and HT suggested PCHD was an early risk indicator for HT. The occurrence of PCHD-3 and PCHD-4 was a strong predictor for END. PCHD-1 is considered to be relatively benign in relation to the 3-month mRS.

## 1. Introduction

Postinterventional cerebral hyperdensity (PCHD) is fairly commonly seen in patients with acute ischemic stroke following intra-arterial treatment [1–3]. Recently, with advances in technology, intravascular stents have been widely used for acute stroke patients; however, there have been few studies on patients with PCHD after mechanical thrombectomy. Previous small-sample studies revealed that PCHD was a strong predictor for final infarction size [4, 5] but was not a risk factor for symptomatic hemorrhage or poor prognosis [5, 6]. This low predictive efficiency may be due to the analysis of only the occurrence of PCHD, rather than the classification of PCHD. Later, Xu et al. found that “metallic hyperdensities” with a CT density of over 90 HU and a larger volume with a bulging contour could predict the occurrence of parenchymal hemorrhage at 24 h [7]. However, there were no further investigations into the effect on clinical prognosis in that study. The classification standard of CT values was formulated in clinical trials evaluating arterial thrombolysis [3], and a later study of intra-arterial revascularization suggested that a CT value of >90 poorly predicted hemorrhagic transformation (HT) with low sensitivity (23%) [8]. There were no further studies of PCHD on clinical prognosis in people with mechanical thrombectomy.

According to the European Cooperative Acute Stroke Study (ECASS) criteria, HT was further divided into four levels [9]. Different types of HT after stroke require corresponding targeted treatment and are closely related to prognosis [10, 11]. However, the correlation between various degrees of HT and PHCD was not clear. Thus, we proposed a new classification of PCHD, which divides PCHD into four levels according to the definition of HT, to provide a more direct and pragmatic early reference sign. The influence and the optimal diagnostic threshold of PCHD subtypes on clinical prognosis were further analyzed.

The aim of our study was to predict HT and clinical prognosis by evaluating the new classification system for PCHD and to provide a theoretical and experimental basis for the formulation of a treatment plan after intra-arterial intervention.

## 2. Materials and Methods

### 2.1. Subjects

The study was approved by the local Ethics Committee, and the requirement for informed consent of every patient was waived.

**Figure 1:**
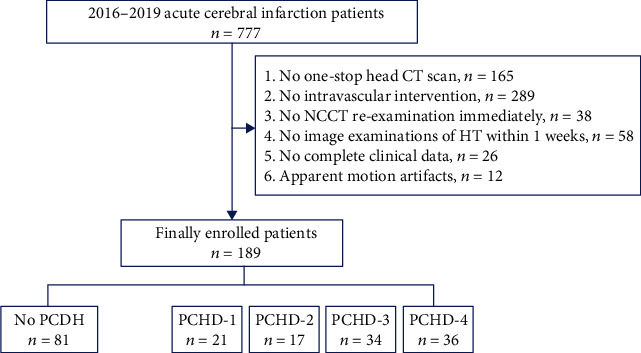
The flowchart of participant recruitment.

The data of 777 patients with acute cerebral infarction from 2016 to 2019 were retrospectively analyzed in the PACS. Among these patients, 189 patients treated with intravascular intervention were enrolled in this study. A flowchart of participant recruitment is shown in Figure 1. According to whether a hyperdensity was found on noncontrast CT scans (NCCT) after intravascular intervention, the patients were divided into a no-PCHD group (*n* = 81) and a PCHD group (*n* = 108). Referring to the new PCHD classification standard, the PCHD group was further divided into PCHD-1, PCHD-2, PCHD-3, and PCHD-4.

The inclusion criteria were as follows: (1) age ≥ 18 years; (2) a clinical diagnosis of acute ischemic stroke at admission; (3) preoperative one-stop head CT scan performed; (4) intravascular intervention performed within the time window; (5) NCCT scan performed immediately after intravascular intervention to observe PCHD; (6) CT reexaminations or SWI examinations performed within 48 h-1 w to observe HT; and (7) availability of complete clinical and imaging data. The exclusion criteria were as follows: (1) vascular malformation; (2) intracranial hemorrhage, infection, or space-occupying lesions; (3) serious heart, lung, or kidney diseases; and (4) obvious motion artifacts.

### 2.2. Clinical Data Acquisition

The following basic clinical characteristics were collected: (1) demographic data, including sex and age; (2) past medical history, history of smoking, hypertension, diabetes, atrial fibrillation, and anticoagulant use; (3) National Institutes of Health Stroke Scale (NIHSS) score at admission; and (4) hematological test data, including total cholesterol (TC), high-density lipoprotein (HDL), low-density lipoprotein (LDL), activated partial thromboplastin time (APTT), and prothrombin time (PT).

Early neurological deterioration (END) was indicated when the NIHSS score increased ≥4 points within 72 h of onset [12].

The mRS score at 3 months: 0-2 scores indicated a good prognosis (no symptoms or mild disability); 3-6 scores indicated a poor prognosis (moderate to severe disability or death) [13, 14].

### 2.3. Image Data Acquisition

Intravascular intervention: for patients without contraindications of venous thrombolysis, mechanical thrombolysis was administered after venous thrombolysis. The time window for anterior circulation strokes was up to 8 h after symptom onset, and that for posterior circulation strokes was 24 h. The time window could be extended appropriately according to ischemic core/penumbra mismatch. The femoral artery was selected for puncture, and the thrombus was removed using a Solitaire stent (Medtronic). Digital subtraction angiography (Allura Xper Fd20 by Philips, Netherlands) was used during mechanical thrombectomy. The sequence included the anterior and lateral positions of each artery. The exposure was automatically adjusted with a delay of 0.5 s, and images of the arterial, parenchymal, and venous phases were collected at a rate of 6 frames per second. Successful recanalization was defined as grade 2b-3 of the modified Thrombolysis in Cerebral Infarction (mTICI) scoring criteria.

Axial NCCT reexamination scan: the SIEMENS Definition AS 128 CT scanner was used. The routine head scan protocol: the tube voltage = 120 kV, the reference current = 400 mA, the actual current can be adjusted by using the combined applications reduce exposure dose 4 dimensions (CARE dose 4D) technology, acquisition matrix = 512 × 512, rebuild field of view (FOV) = 300 mm × 300 mm, layer thickness = 1 mm, and interslice gap = 0. The emergency head scan protocol: the tube voltage = 120 kV, the reference current = 400 mA, the actual current can be adjusted by using the CARE dose 4D technology, acquisition matrix = 512 × 512, rebuild FOV = 300 mm × 300 mm, and pitch = 0.9 mm.

SWI: TR = Minimum, TE = 24.3 ms, FOV = 200 × 200 mm, slice thickness = 1.6 mm, and interslice gap = 0.

HT was defined as a hyperdensity that persisted or was extended on CT reexamination within 1 week or on a susceptibility-weighted imaging sequence (SWI) that showed a low signal in the infarct range. HT was classified according to the ECASS definition. HI means hemorrhagic infarction, and pH means parenchymal hemorrhage.

- HI-1: small confluent petechiae without a space-occupying effect or nonsolid
- HI-2: more confluent petechiae without a space-occupying effect or nonsolid
- pH-1: less than 30% of the infarcted area has a mild mass effect
- pH-2: greater than 30% of the infarcted area has a significant space-occupying effect

**Figure 2:**
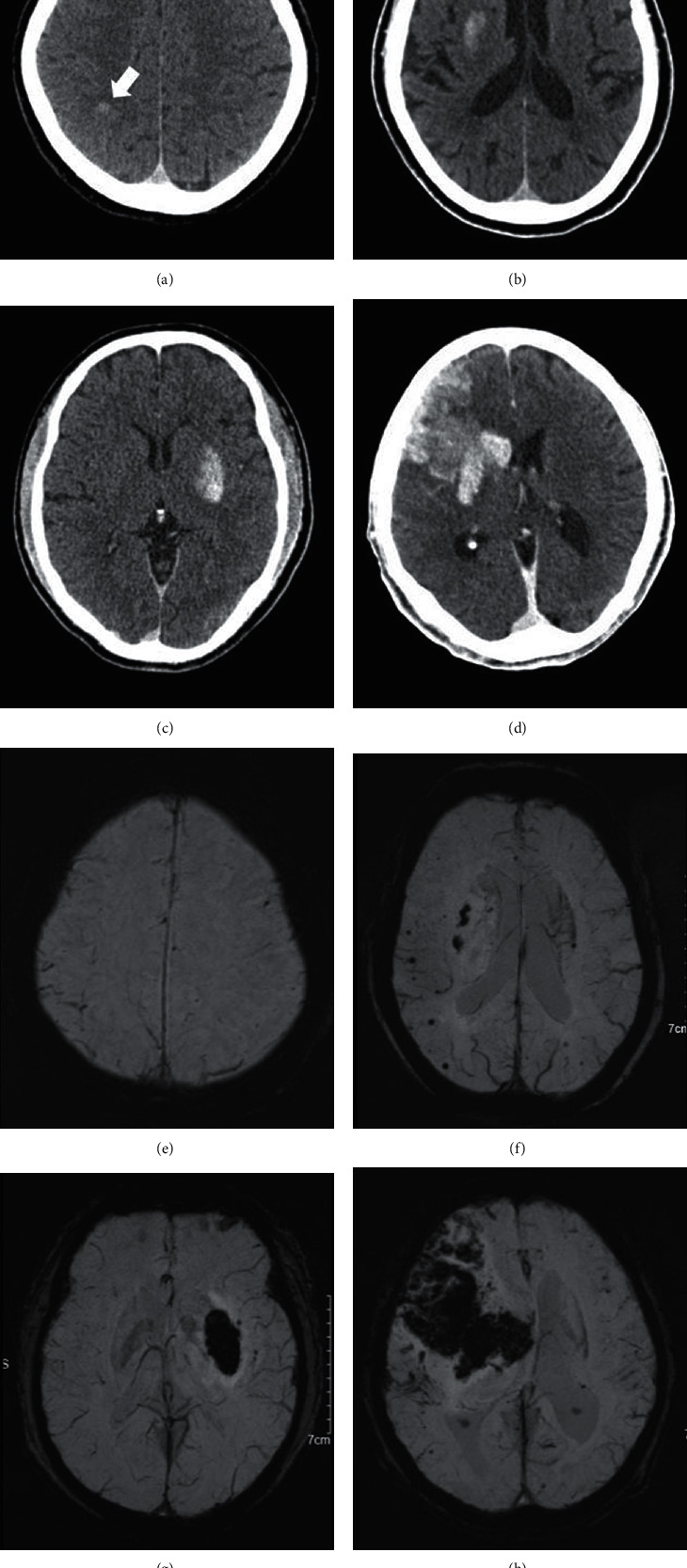
The upper row of images (a–d) show the four types of PCHD, and the lower row of images (e–h) show the prognosis of TH in the corresponding four patients. PCHD-1 showing small area of hyperdensity (the white arrow) without space-occupying effects (a), no TH in follow-up SWI image (e). PCHD-2 showing more confluent hyperdensity at the right basal ganglia without space-occupying effects (b), HI-1 was observed during follow-up (f). PCHD-3 showing hyperdensity at the right basal ganglia accompany the ipsilateral narrow sulci (c), pH-1 was observed during follow-up (g). PCHD-4 showing hyperdensity with a significant space-occupying effect (d), pH-2 was observed during follow-up (h).

PCHD was defined by visually distinctive parenchymal hyperdense areas diagnosed within 24 h after intravascular intervention, with a diameter of at least 0.1 cm^2^ and an increased density of at least 5 HU (HUmax) compared to the unaffected contralateral area. The example diagram of PCHD is shown in Figure 2.

- PCHD-1: small area of hyperdensity without space-occupying effects
- PCHD-2: more confluent hyperdensity without space-occupying effects
- PCHD-3: less than 30% of the infarcted area has a mild mass effect
- PCHD-4: greater than 30% of the infarcted area has a significant space-occupying effect

Imaging indexes (the Alberta Stroke Program Early CT (ASPECT) score and the classification of PCHD and HT) were evaluated by two neuroradiologists (Dr. Shao, who has 3 years of experience, and Dr. He, who has 13 years of experience). The two doctors first evaluate independently and finally reach a consensus through discussion where there is disagreement.

### 2.4. Statistical Analysis

Statistical analysis was performed with SPSS 20.0 (IBM, Chicago, IL, United States). Comparisons of the basic clinical characteristics were conducted with a *t*-test, the chi-square test, or the Mann–Whitney *U* test, depending on the circumstances. The Crosstabs test and ANOVA were used for the four subtypes of PCHD. The intraclass correlation coefficient was calculated to evaluate the consistency between the two observers. The Spearman rank correlation test was used to analyze the correlation between PCHD and HT, and *r* > 0.75 was considered to be a good correlation. Univariate logistic regression analysis was used to explore the relationship between dependent variables and independent variables. A forest plot was established to show the relationship. Then, through multivariate logistic regression analysis, the independent factors that were associated with END and mRS were further identified. Finally, a receiver-operating characteristic (ROC) curve was constructed. The area of curves (AUC) was used to evaluate the diagnostic efficacy of the independent risk factors for END and mRS at 3 months. Cutoff value was used to identify the threshold that predicts clinical outcomes. Statistical significance was set at *p* ≤ 0.05.

## 3. Results

### 3.1. Demographic and Clinical Characteristics

A total of 189 patients were included in this study, with 81 (42.9%) patients in the no-PCHD group and 108 (57.1%) patients in the PCHD group. The incidence of HT in our study was 50.8% (96/189), HI = 31.2% (59/189), and pH = 19.6% (37/189).

As shown in Table 1, there were significant differences in the history of hypertension, APTT, the NIHSS score at admission, and the ASPECT score between the no-PCHD group and the PCHD group (*p* < 0.05). There was no significant difference in the location of occluded artery between the two groups (*p* > 0.05).

As shown in Table 2, there were significant differences in the ASPECT score among the four PCHD subgroups (*p* = 0.009). The CT value of the high-density PCHD-4 group was higher than that of the PCHD-1 and PCHD-2 groups (*p* = 0.002), but no difference was found between these three subgroups and the PCHD-3 group. Patients in the PCHD-1/2 group lacked intraventricular hyperdensity, which was significantly different from that in PCHD-3/4 group (*p* = 0.002). Moreover, there were significant differences in clinical prognosis among the four groups (HT, END, and mRS, all *p* < 0.05).

The two observers performed radiographic evaluations with a high degree of consistency. The intraclass correlation coefficients of the ASPECT score, PCHD and HT were 0.950, 0.986, and 0.978, respectively, all *p* < 0.01.

### 3.2. The Correlation between PCHD and HT

**Figure 3:**
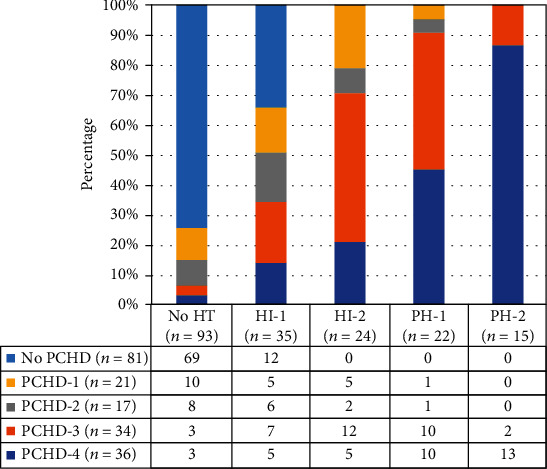
The correlation between the classifications of PCHD and HT.

The incidence of HT was 14.8% (12/81) in the no-PCHD group and 77.8% (84/108) in the PCHD group. As shown in Figure 3, in the no-PCHD group, 69 of the patients did not present HT and 12 of the patients presented HI-1; however, no patients presented HI-2, pH-1, and pH-2. All patients with subsequent pH-2 were in the PCHD-3 and PCHD-4 subgroup in the early stage (PCHD − 3 = 86.67%, PCHD − 4 = 13.33%). The Spearman rank correlation analysis showed that there was a good correlation between PCHD and HT (*r* = 0.751, *p* < 0.01).

### 3.3. Predicting Early Neurological Deterioration (END)

**Figure 4:**
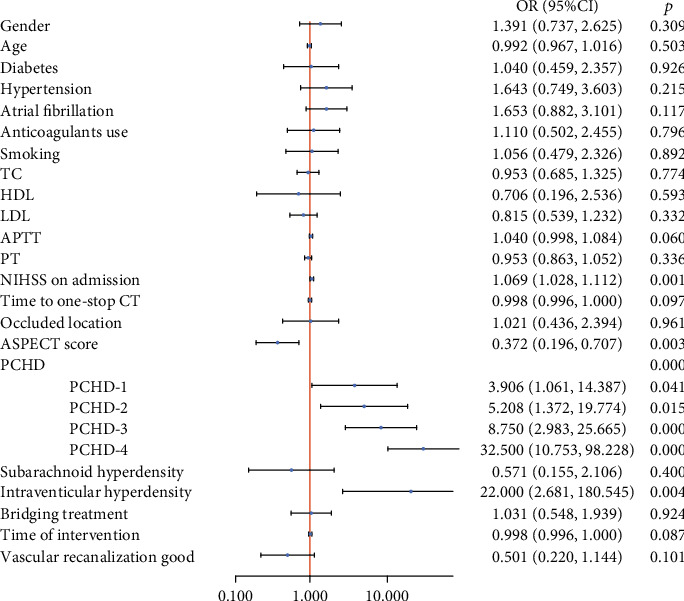
Univariate predictors of END.

**Figure 5:**
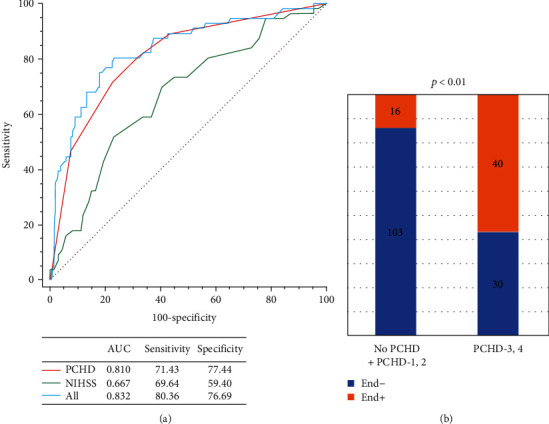
Evaluation of the accuracy of END. (a) ROC curves for the NIHSS score at admission and PCHD in predicting END. (b) The probability of END in the high-risk group was significantly higher than that in the low-risk group, and the optimal diagnostic cutoff value was PCHD > 2.

The image and clinical data of END- and END+ group were shown in Table s1. The univariate analysis revealed there were significant differences in the NIHSS score, the ASPECT score, PCHD classification, and interventricular hyperdensity between the two groups (*p* < 0.05, illustrated Figure 4). After the multivariate regression analysis, the NIHSS score at admission and PCHD were independent factors for END (*p* = 0.015, *p* < 0.001, respectively), as in Table 3.  Figure 5 showed that AUC of PCHD was 0.810, and the optimal diagnostic cutoff value was PCHD > 2. The AUC of the NIHSS score at admission was 0.667. The AUC of the combined model was 0.832.

### 3.4. Predicting the mRS Score at 3 Months

**Figure 6:**
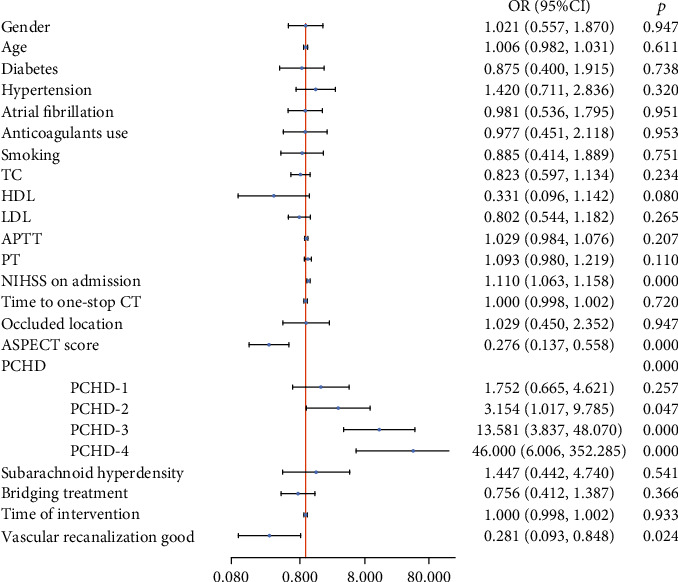
Univariate predictors of the mRS score at 3 months.

**Figure 7:**
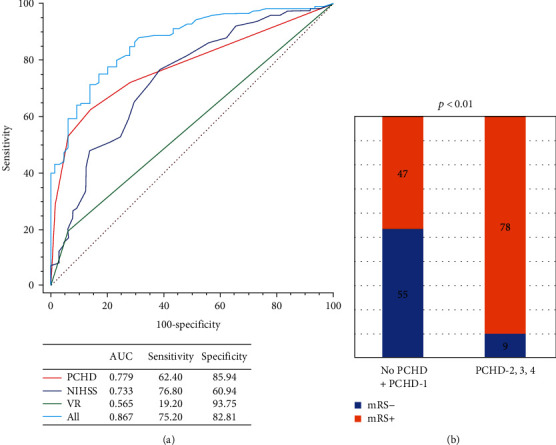
Evaluation of the accuracy of the mRS score at 3 months. (a) ROC curves for good vascular recanalization (VR), the NIHSS score at admission, and PCHD in predicting the mRS+ (≥3 score) at 3 months. (b) The probability of the mRS+ at 3 months in the high-risk group was significantly higher than that in the low-risk group, and the optimal diagnostic cutoff value was PCHD > 1.

The image and clinical data of mRS- and mRS+ group were shown in Table s2. Univariate analysis revealed that the NIHSS score at admission, the ASPECT score, PCHD classification, and good vascular recanalization were associated with the 3-month mRS (all *p* < 0.05), as shown in Figure 6. The multivariate analysis revealed that the NIHSS score, PCHD, and good vascular recanalization were independently associated with the mRS score at 3 months (all *p* < 0.05). Good vascular recanalization was a protective factor (OR = 0.256, 0.070-0.933), but the predictive efficiency was lower (AUC = 0.565), as in Table 4.  Figure 7 showed that AUC of PCHD was 0.779, and the optimal diagnostic cutoff value was PCHD > 1. The AUC of the NIHSS score at admission, good vascular recanalization, and the combined model were 0.733, 0.565, and 0.867, respectively.

## 4. Discussion

This study shows that there was a good correlation between the new classification system for PCHD and HT. The patients with PCHD-3 and PCHD-4 were more likely to have early neurological deterioration. The 3-month mRS was poorer in patients with PCHD classified as PCHD-2, PCHD-3, and PCHD-4, but PCHD-1 was considered to be relatively benign.

In our study, the incidence of PCHD after intra-arterial reperfusion therapy was 57.1%, which was consistent with the incidence of PCHD reported by others (32.9% to 84.2%) [1–3, 15]. The incidence of HT in our study was 50.8% (HI = 31.2%; pH = 19.6%). The incidence of pH was higher than that in several published multicenter randomized clinical trials, ranging from 3.6% to 11% [16–20]. However, our rate was determined from real-world data after mechanical thrombectomy. The lack of strict control over the time window, which was extended according to ischemic core/penumbra mismatch, may be one of the reasons for this discrepancy. Another reason may be the use of SWI to detect HT, which may result in a visual overestimation of hematoma size due to susceptibility effects [21].

This study is the first to classify PCHD according to the ECASS definition in an attempt to develop a simpler and more intuitive method to predict different types of HT. Our results showed that there was a good correlation between the subtypes of PCHD and HT. Therefore, we propose that both of these occurrences have a similar pathological basis, resulting from microvascular damage and increased permeability of the BBB. A scholar proposed that when the BBB loss is small, the main extravasation content is contrast agent, which will be absorbed during follow-up. Larger red blood cells also leak out when BBB breakdown is evident. A large number of red blood cells in the brain parenchyma can cause a significant mass effect, resulting in poor clinical prognosis [15, 22, 23]. The treatment and clinical prognosis of different types of HT vary, and this study provides a theoretical basis for selecting the appropriate subsequent treatment through the early evaluation of PCHD. In this study, we also found that the CT values in the PCHD-3 group were not well differentiated from those in the other three groups, which may explain the low predictive ability of CT values for HT [8].

Previous studies have shown that the presence of hyperdensity in patients with arterial thrombolysis has no significant effect on the clinical prognosis [5, 6]. There were no further studies of PCHD on clinical prognosis in patients with mechanical thrombectomy. In our study, hyperdensity was classified into more detailed grades, and our results suggested that there were different cutoff points for early or long-term clinical prognosis. The finding of space-occupying, intervention-related PCHD 3 and PCHD-4 was associated with a high likelihood of END. This suggests that in patients with large-vessel infarction, the accumulation of mass effects may lead to further deterioration of neurological function [23].

PCHD and the NIHSS score at admission were independent risk factors for mRS at 3 months, while good vascular recanalization was a protective factor. Our results suggested that PCHD-2, PCHD-3, and PCHD-4 had a negative impact on the long-term functional outcome compared with PCHD-1. Our finding was similar to that of a previous study [11], which demonstrated the influence of hemorrhagic transformation on long-term prognosis after thrombolysis, confirming the greater clinical relevance of the new classification system for PCHD and HT.

### 4.1. Limitations

This study was a single-center retrospective analysis. HT was diagnosed by two imaging methods, follow-up CT or SWI. Although both diagnostic methods have been used in other studies, some bias may still be present [24, 25].

## 5. Conclusions

There was a good correlation between HT and PCHD after intravascular intervention in ischemic stroke patients. PCHD was an independent factor for END and the mRS score at 3 months with different cutoff points. The new classification system for PCHD was suggested to have some clinical significance for predicting both the subtypes of HT and clinical prognosis.

## Figures and Tables

**Table 1:** Basic characteristics of the no-PCHD and PCHD groups.

	No-PCHD group (*n* = 81)	PCHD group (*n* = 108)	*p*
Age	70.19 ± 12.74	70.27 ± 12.18	0.964
Sex (male; %)	43; 53.1%	61; 56.5%	0.642
History of smoking	15; 18.5%	21; 19.4%	0.873
History of hypertension	56; 69.1%	88; 81.5%	0.049
History of atrial fibrillation	32; 39.5%	56; 51.9%	0.092
History of diabetes	11; 13.6%	22; 20.4%	0.224
History of anticoagulant use	11; 13.6%	24; 22.2%	0.130
TC	3.86 ± 0.97	3.82 ± 0.94	0.774
HDL	1.06 ± 0.24	1.07 ± 0.26	0.668
LDL	2.32 ± 0.78	2.20 ± 0.77	0.295
APTT	27.41 ± 5.48	32.76 ± 24.52	0.030
PT	13.17 ± 3.39	13.35 ± 4.01	0.744
NIHSS score at admission	19.27 ± 9.61	22.86 ± 7.82	0.007
ASPECT score (<6 scores)	17; 21.0%	56; 51.9%	<0.001
Location of occlusion			0.900
ICA isolated or in tandem with MCA	11; 13.6%	15; 13.9%	
MCA isolated	56; 69.1%	77; 71.3%	
Posterior circulation	14; 17.3%	16; 14.8%	

TC: total cholesterol; HDL: high-density lipoprotein; LDL: low-density lipoprotein; APTT: activated partial thromboplastin time; PT: prothrombin time; ICA: internal carotid artery; MCA: middle cerebral artery.

**Table 2:** Demographic and clinical characteristics of the four subtypes of PCHD.

	PCHD-1 (*n* = 21)	PCHD-2 (*n* = 17)	PCHD-3 (*n* = 34)	PCHD-4 (*n* = 36)	*p*
Age	68.90 ± 15.16	70.29 ± 9.71	71.39 ± 13.71	70.27 ± 12.18	0.900
Sex (male; %)	8; 38.1%	10; 58.8%	22; 64.7%	21; 58.3%	0.271
Smoking	3; 14.3%	3; 17.6%	6; 17.6%	9; 25.0%	0.763^f^
Hypertension	17; 81.0%	15; 88.2%	27; 79.4%	29; 80.6%	0.875^f^
Atrial fibrillation	11; 52.4%	8; 47.1%	19; 55.9%	18; 50.0%	0.934
Diabetes	2; 9.5%	1; 5.9%	10; 29.4%	9; 25.0%	0.082^f^
Anticoagulant use	5; 23.8%	5; 29.4%	11; 32.4%	3; 8.3%	0.063^f^
TC	3.77 ± 0.87	3.77 ± 1.39	3.80 ± 0.72	3.89 ± 0.95	0.956
HDL	1.11 ± 0.17	1.02 ± 0.27	1.05 ± 0.25	1.09 ± 0.30	0.633
LDL	2.15 ± 0.75	2.25 ± 1.10	2.11 ± 0.52	2.28 ± 0.82	0.802
APTT	27.83 ± 4.65	34.65 ± 31.43	37.34 ± 37.03	30.42 ± 6.24	0.487
PT	13.10 ± 1.66	11.95 ± 3.20	13.59 ± 4.27	13.94 ± 4.94	0.390
Time of onset (h)	3.65 ± 2.47	4.64 ± 3.20	4.10 ± 1.61	4.18 ± 2.45	0.647
NIHSS at admission	24.05 ± 7.72	20.71 ± 5.44	21.47 ± 9.44	24.50 ± 6.90	0.219
ASPECT score	5; 23.8%	8; 47.1%	24; 70.6%	19; 52.8%	0.009
OP (AC; %)	16; 76.2%	17; 100.0%	28; 82.4%	31; 86.1%	0.077^f^
CT value (HU)	65.38 ± 35.68	61.88 ± 27.56	83.56 ± 47.21	148.53 ± 156.52	0.002^∗^
Subarachnoid HD	4; 19.0%	1; 5.9%	5; 14.7%	4; 11.1%	0.628^f^
Intraventricular HD	0; 0.0%	0; 0.0%	1; 2.9%	8; 22.2%	0.002^f^
Operation time (h)	5.38 ± 2.47	5.89 ± 2.60	5.19 ± 1.70	5.70 ± 2.78	0.726
VR (good, %)	19; 90.5%	15; 88.2%	30; 88.2%	28; 77.8%	0.510^f^
Bridging treatment	6; 28.6%	9; 52.9%	15; 44.1%	14; 38.9%	0.467
HT	11; 52.4.0%	9; 52.9%	31; 91.2%	33; 91.7%	<0.001^f^
END	5; 23.8%	5; 29.4%	14; 41.2%	26; 72.2%	0.001
3-month mRS	12; 57.1%	12; 70.6%	31; 91.2%	35; 97.2%	<0.001^f^

TC: total cholesterol; HDL: high-density lipoprotein; LDL: low-density lipoprotein; APTT: activated partial thromboplastin time; PT: prothrombin time; OP: occluded position; AC: anterior circulation; HD: hyperdensity; VR: vascular recanalization; HT: hemorrhagic transformation; END: early neurological deterioration; f: Fisher's exact test; ^∗^: the CT value of the high-density PCHD-4 group was higher than that of the PCHD-1 and PCHD-2 groups, but no difference was found between these three subgroups and the PCHD-3 group.

**Table 3:** Stepwise logistic regression analysis predicting END.

	Univariate logistic regression		Multivariate logistic regression	
	OR (95% CI)	*p*	OR (95% CI)	*p*
Sex	1.391 (0.737, 2.625)	0.309		
Age	0.992 (0.967, 1.016)	0.503		
Diabetes	1.040 (0.459, 2.357)	0.926		
Hypertension	1.643 (0.749, 3.603)	0.215		
Atrial fibrillation	1.653 (0.882, 3.101)	0.117		
Anticoagulant use	1.110 (0.502, 2.455)	0.796		
Smoking	1.056 (0.479, 2.326)	0.892		
TC	0.953 (0.685, 1.325)	0.774		
HDL	0.706 (0.196, 2.536)	0.593		
LDL	0.815 (0.539, 1.232)	0.332		
APTT	1.040 (0.998, 1.084)	0.060		
PT	0.953 (0.863, 1.052)	0.336		
NIHSS at admission	1.069 (1.028, 1.112)	0.001	1.061 (1.011, 1.113)	0.015
Time to one-stop CT	0.998 (0.996, 1.000)	0.097		
Occluded location	1.021 (0.436, 2.394)	0.961		
ASPECT score	0.372 (0.196, 0.707)	0.003	/	/
PCHD		<0.001		<0.001
PCHD-1	3.906 (1.061, 14.387)	0.041	3.197 (0.847, 12.070)	0.086
PCHD-2	5.208 (1.372, 19.774)	0.015	5.362 (1.386, 20.746)	0.015
PCHD-3	8.750 (2.983, 25.665)	<0.001	8.581 (2.853, 25.804)	<0.001
PCHD-4	32.500 (10.753, 98.228)	<0.001	28.240 (9.206, 86.626)	<0.001
Subarachnoid HD	0.571 (0.155, 2.106)	0.400		
Intraventricular HD	22.000 (2.681, 180.545)	0.004	/	/
Bridging treatment	1.031 (0.548, 1.939)	0.924		
Time of intervention	0.998 (0.996, 1.000)	0.087		
VR (good)	0.501 (0.220, 1.144)	0.101		

TC: total cholesterol; HDL: high-density lipoprotein; LDL: low-density lipoprotein; APTT: activated partial thromboplastin time; PT: prothrombin time; HD: hyperdensity; VR: vascular recanalization.

**Table 4:** Stepwise logistic regression analysis predicting mRS score at 3 months.

	Univariate logistic regression		Multivariate logistic regression	
	OR (95% CI)	*p*	OR (95% CI)	*p*
Sex	1.021 (0.557, 1.870)	0.947		
Age	1.006 (0.982, 1.031)	0.611		
Diabetes	0.875 (0.400, 1.915)	0.738		
Hypertension	1.420 (0.711, 2.836)	0.320		
Atrial fibrillation	0.981 (0.536, 1.795)	0.951		
Anticoagulant use	0.977 (0.451, 2.118)	0.953		
Smoking	0.885 (0.414, 1.889)	0.751		
TC	0.823 (0.597, 1.134)	0.234		
HDL	0.331 (0.096, 1.142)	0.080		
LDL	0.802 (0.544, 1.182)	0.265		
APTT	1.029 (0.984, 1.076)	0.207		
PT	1.093 (0.980, 1.219)	0.110		
NIHSS at admission	1.110 (1.063, 1.158)	<0.001	1.108 (1.054, 1.165)	<0.001
Time to one-stop CT	1.000 (0.998, 1.002)	0.720		
Occlusion location	1.029 (0.450, 2.352)	0.947		
ASPECT score	0.276 (0.137, 0.558)	<0.001	/	/
PCHD		<0.001		<0.001
PCHD-1	1.752 (0.665, 4.621)	0.257	1.272 (0.438, 3.695)	0.658
PCHD-2	3.154 (1.017, 9.785)	0.047	3.250 (0.988, 10.688)	0.052
PCHD-3	13.581 (3.837, 48.070)	<0.001	18.778 (4.801, 73.450)	<0.001
PCHD-4	46.000 (6.006, 352.285)	<0.001	36.294 (4.593, 286.822)	0.001
Subarachnoid HD	1.447 (0.442, 4.740)	0.541		
Bridging treatment	0.756 (0.412, 1.387)	0.366		
Time of intervention	1.000 (0.998, 1.002)	0.933		
VR (good)	0.281 (0.093, 0.848)	0.024	0.245 (0.066, 0.908)	0.035

TC: total cholesterol; HDL: high-density lipoprotein; LDL: low-density lipoprotein; APTT: activated partial thromboplastin time; PT: prothrombin time; HD: hyperdensity; VR: vascular recanalization.

## Data Availability

Data can be accessed by inquiring the corresponding author.

## Supplementary Materials

Supplementary Materials The following supplementary materials are available for this paper: image and clinical data of END- and END+ group (Table s1), and image and clinical data of mRS- and mRS+ group (Table s2).
