# Supplementary material for: A New Classification System for Postinterventional Cerebral Hyperdensity: The Influence on Hemorrhagic Transformation and Clinical Prognosis in Acute Stroke
Source: Neural Plast. 2021 Nov 23;2021:6144304. doi: 10.1155/2021/6144304 (PMC8632469; doi:10.1155/2021/6144304)
Supplement: Supplementary Materials — The following supplementary materials are available for this paper: image and clinical data of END- and END+ group (Table s1), and image and clinical data of mRS- and mRS+ group (Table s2). [file 6144304.f1.docx]

| Table s1. Image and clinical data of END- and END+ group | | | |
| --- | --- | --- | --- |
|  | **END- (n=133)** | **END+ (n=56)** | ***p*** |
| **Clinical data at baseline** |  |  |  |
| Age | 70.62±12.16 | 69.30±12.99 | 0.505 |
| Gender (male, %) | 70(52.6%) | 34(60.7%) | 0.308 |
| History of smoking | 25(18.8%) | 11(19.6%) | 0.892 |
| History of hypertension | 98(73.7%) | 46(82.1%) | 0.213 |
| History of atrial fibrillation | 57(42.9%) | 31(55.4%) | 0.116 |
| History of diabetes | 23(17.3%) | 10(17.9%) | 0.926 |
| History of anticoagulants use | 24(18.0%) | 11(19.6%) | 0.796 |
| TC | 3.85±1.00 | 3.81±0.84 | 0.776 |
| HDL | 1.07±0.24 | 1.05±0.26 | 0.595 |
| LDL | 2.29±0.79 | 2.17±0.74 | 0.333 |
| APTT | 27.94±6.04 | 36.47±33.13 | 0.061 |
| PT | 13.45±4.04 | 12.87±2.95 | 0.332 |
| NIHSS at admission | 19.89±8.54 | 24.71±8.50 | <0.001 |
| **Image data** |  |  |  |
| Time to one-stop CT (min) | 261.89±168.08 | 220.43±112.80 | 0.092 |
| Occluded location (AC) | 112; 84.2% | 47; 83.9% | 0.961 |
| ASPECT score (≥6 scores) | 91(68.4%) | 25(44.6%) | 0.002 |
| PCHD |  |  | <0.001 |
| No PCHD | 75(56.4%) | 6(10.7%) |  |
| PCHD-1 | 16(12.0%) | 5(8.9%) |  |
| PCHD-2 | 12(9.0%) | 5(8.9%) |  |
| PCHD-3 | 20 (15.0%) | 14 (25.0%) |  |
| PCHD-4 | 10 (7.5%) | 26 (46.4%) |  |
| Subarachnoid hyperdensity | 12(9.0%) | 3(5.4%) | 0.559^f^ |
| Intraventricular hyperdensity | 1(0.8%) | 8(14.3%) | <0.001^f^ |
| **Treatment** |  |  |  |
| Prior intravenous thrombolysis | 56(42.1%) | 24(42.9%) | 0.924 |
| Time of intervention (min) | 345.75±167.38 | 303.27±111.48 | 0.083 |
| VR (good, %) | 117(88.0%) | 44(78.6%) | 0.097 |
| TC: Total cholesterol; HDL: high-density lipoprotein; LDL: Low-density lipoprotein; APTT: activated partial thromboplastin time; PT: prothrombin time; AC: anterior circulation, VR: vascular recanalization. f：Fisher's Exact Test; | | | |

| Table s2. Image and clinical data of mRS- and mRS+ group. | | | |
| --- | --- | --- | --- |
|  | **mRS- (n=64)** | **mRS + (n=125)** | ***p*** |
| **Clinical data at baseline** |  |  |  |
| Age | 69.59±12.08 | 70.56±12.58 | 0.613 |
| Gender (male, %) | 35 (54.7%) | 69 (55.2%) | 0.947 |
| History of smoking | 13 (20.3%) | 23 (18.4%) | 0.751 |
| History of hypertension | 46 (71.9%) | 98 (78.4%) | 0.319 |
| History of atrial fibrillation | 30 (46.9%) | 58 (46.4%) | 0.951 |
| History of diabetes | 12 (18.8%) | 21 (16.8%) | 0.738 |
| History of anticoagulants use | 12 (18.8%) | 23 (18.4%) | 0.953 |
| TC | 3.95±0.945 | 3.78±0.951 | 0.234 |
| HDL | 1.11±0.262 | 1.04±0.237 | 0.078 |
| LDL | 2.34±0.771 | 2.21±0.776 | 0.266 |
| APTT | 27.10, 6.30 | 27.90, 7.95 | 0.143 |
| PT | 12.10, 1.58 | 12.70, 2.10 | 0.003 |
| NIHSS at admission | 16.766±8.234 | 23.656±8.151 | <0.001 |
| **Image data** |  |  |  |
| Time to one-stop CT (min) | 207.00, 164.75 | 240.00, 137.50 | 0.242 |
| Occluded location (AC) | 10 (15.6%) | 20 (16.0%) | 0.947 |
| ASPECT score (≥6 scores) | 51 (79.7%) | 65 (52.0%) | <0.001 |
| PCHD |  |  | <0.001 |
| No PCHD | 46 (71.9%) | 35 (28.0%) |  |
| PCHD-1 | 9 (14.1%) | 12 (9.6%) |  |
| PCHD-2 | 5 (7.8%) | 12 (9.6%) |  |
| PCHD-3 | 3 (4.7%) | 31 (24.8%) |  |
| PCHD-4 | 1 (1.6%) | 35 (28.0%) |  |
| Subarachnoid hyperdensity | 4 (6.2%) | 11 (8.8%) | 0.539 |
| Intraventricular hyperdensity | 0 (0.0%) | 9 (7.2%) | 0.028 |
| **Treatment** |  |  |  |
| Prior intravenous thrombolysis | 30 (46.9%) | 50 (40.0%) | 0.365 |
| Time of intervention (min) | 298.00, 169.00 | 306.00, 168.50 | 0.779 |
| VR (good, %) | 60 (93.8%) | 101 (80.8%) | 0.018 |
| TC: Total cholesterol; HDL: high-density lipoprotein; LDL: Low-density lipoprotein; APTT: activated partial thromboplastin time; PT: prothrombin time; AC: anterior circulation, VR: vascular recanalization. | | | |
